# TRPM2 Channel-Mediated ROS-Sensitive Ca^2+^ Signaling Mechanisms in Immune Cells

**DOI:** 10.3389/fimmu.2015.00407

**Published:** 2015-08-07

**Authors:** Sharifah Alawieyah Syed Mortadza, Lu Wang, Dongliang Li, Lin-Hua Jiang

**Affiliations:** ^1^School of Biomedical Sciences, University of Leeds, Leeds, UK; ^2^Key Laboratory of Brain Research of Henan Province, Department of Physiology and Neurobiology, Xinxiang Medical University, Xinxiang, China

**Keywords:** TRPM2, reactive oxygen species, Ca^2+^ signaling, immune cell functions, inflammatory diseases

## Abstract

Transient receptor potential melastatin 2 (TRPM2) proteins form Ca^2+^-permeable cationic channels that are potently activated by reactive oxygen species (ROS). ROS are produced during immune responses as signaling molecules as well as anti-microbial agents. ROS-sensitive TRPM2 channels are widely expressed in cells of the immune system and located on the cell surface as a Ca^2+^ influx pathway in macrophages, monocytes, neutrophils, lymphocytes, and microglia but preferentially within the lysosomal membranes as a Ca^2+^ release mechanism in dendritic cells; ROS activation of the TRPM2 channels, regardless of the subcellular location, results in an increase in the intracellular Ca^2+^ concentrations. Recent studies have revealed that TRPM2-mediated ROS-sensitive Ca^2+^ signaling mechanisms play a crucial role in a number of processes and functions in immune cells. This mini-review discusses the recent advances in revelation of the various roles the TRPM2 channels have in immune cell functions and the implications in inflammatory diseases.

## Introduction

Cells of the immune system, including monocytes, macrophages, dendritic cells (DCs), neutrophils, T and B lymphocytes, and natural killer cells, play a critical role in orchestrating both innate and adaptive immune responses to microbial pathogens, environment irritants, and danger molecules released from damaged cells. Microglia represents the residual macrophages in the central nervous system (CNS) responsible for immune responses to nerve damages. Intracellular Ca^2+^ is a universal and vital signaling molecule in almost every mammalian cell. Several Ca^2+^ signaling mechanisms are well known in immune cells. Activation of the T-cell receptor, B-cell receptor, and Fc receptors, which are coupled to phospholipase C γ (PLCγ), or the PLCβ-coupled chemokine receptors, generates inositol-1,4,5-trisphosphate (IP_3_) to activate the IP_3_ receptors and release Ca^2+^ from the endoplasmic reticulum (ER). Reduction in the ER Ca^2+^ elicits store-operated Ca^2+^ entry through Ca^2+^ release-activated Ca^2+^ channels to stimulate Ca^2+^ influx and increase the intracellular Ca^2+^ concentrations ([Ca^2+^]_i_) (1, 2). Purinergic receptors for extracellular nucleotides such as ATP, comprising P2X and P2Y subfamilies, are another set of recognized immune Ca^2+^ signaling mechanisms. Several P2Y receptors, like chemokine receptors, are cascaded to the PLCβ-IP_3_ receptor signaling pathway, whereas P2X receptors are ligand-gated Ca^2+^-permeable channels all mediating Ca^2+^ influx (3, 4). The P2X7 receptor was formerly named P2Z receptor in immune cells for its low sensitivity to ATP and its intriguing ability to induce formation of large and cytolytic pores (5–7).

**Figure 1:**
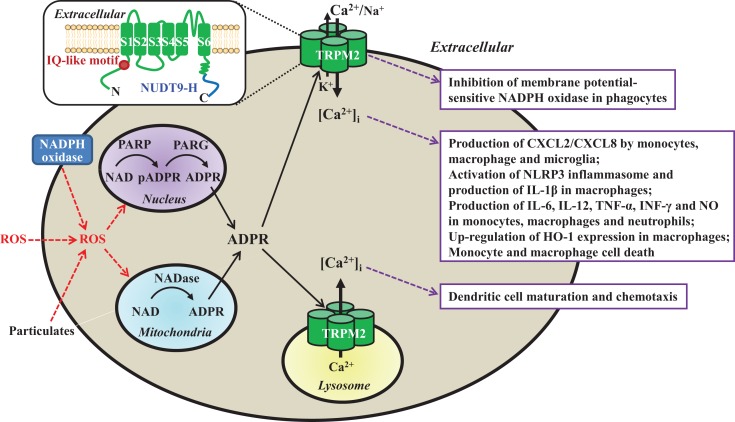
**Subcellular localization, activation mechanisms, and functional roles of the TRPM2 channels in immune cells**. TRPM2 channels are present as a Ca^2+^-permeable cationic channel on the immune cell surface with the exception of dendritic cell, where they are localized in the lysosomal membranes as a Ca^2+^ release channel. TRPM2 channels are activated by intracellular ADPR and Ca^2+^ via upon binding to the C-terminal NUDT9-H domain and N-terminal IQ-like calmodulin-binding motif, respectively (highlighted in the *insert*), and by ROS (e.g., H_2_O_2_) through the mechanisms engaging PARP/PARG in the nucleus or NADase in the mitochondria to generate ADPR from NAD. ROS are generated by phagocytes via NADPH oxidases, or by mitochondria in response to particulates as the NLRP3 inflammasome activation signals (e.g., lipids, silica, or alum). Activation of the TRPM2 channels mediates K^+^ efflux and Ca^2+^/Na^+^ influx to induce membrane depolarization and inhibits membrane potential-sensitive NADPH oxidases to limit ROS production by phagocytes (box 1). TRPM2-mediated Ca^2+^ influx triggers multiple-step intracellular signaling pathways (not depicted) in various immune cells, leading to production of chemokine CXCL8/CXCL2 and proinflammatory cytokines/mediators, up-regulation of HO-1 expression, and cell death (box 2). Finally, TRPM2-mediated lysosomal Ca^2+^ release is required for chemokine-induced dendritic cell maturation and chemotaxis (box 3). *Insert*: a schematic presentation of one TRPM2 subunit in the tetrameric channel, which is composed of six transmembrane segments (S1–S6) and a pore-forming loop between the S5 and S6, and intracellular N- and C-termini. ROS, reactive oxygen species; NAD, nicotinamide adenine dinucleotide; ADPR, ADP-ribose; PARP, poly(ADP-ribose) polymerase; PARG; poly(ADP-ribose) glycohydrolase; pADPR, poly(ADP-ribose) moiety; NADPH oxidase, nicotinamide adenine dinucleotide phosphate oxidase; CXCL, C-X-C ligand; IL, interleukin; TNF-α, tumor necrosis factor-α; INF-γ, interferon γ; NO, nitric oxide; HO-1, heme oxygenase-1.

Mammalian cells express a large family of transient receptor potential (TRP) proteins, which are commonly divided based on sequence relatedness into TRPC (canonical), TRPV (vanilloid), TRPM (melastatin), TRPA (ankyrin), TRPP (polycystin), and TRPML (mucolipin), but all have the same membrane arrangement and form cationic channels with a majority permeating Ca^2+^ (8, 9). The recently determined atomic structures of the TRPV1 channel define the tetrameric assembly (10); each subunit comprise six transmembrane segments (S1–S6) and intracellular N- and C-termini, and the ion permeation pathway is made of the S5, S6, and re-entrant loop between them from each of the four subunits. The mammalian transient receptor potential melastatin 2 (TRPM2) genes, cloned so far from human, rat, and mouse, encode proteins of ~1300 amino acid residues and ~171 kDa (11). Two seminal studies in 2001 are the first to show that TRPM2 proteins form Ca^2+^-permeable cationic channels gated by intracellular ADP-ribose (ADPR) upon binding to the unique NUDT9 homology (NUDT9-H) domain in the distal C-terminus (12, 13) (Figure 1). Studies shortly demonstrated that TRPM2 channels are potently activated by reactive oxygen species (ROS) (14–16), mainly through ADPR-generating mechanisms engaging poly(ADP-ribose) polymerase (PARP) and poly(ADP-ribose) glycohydrolase in the nucleus or NADase in the mitochondria (Figure 1). Subsequent studies have revealed that intracellular Ca^2+^ can activate the TRPM2 channels independently via an IQ-like calmodulin-binding motif in the N-terminus (17) (Figure 1).

It is well recognized that immune cells generate ROS as anti-microbial agents and also signaling molecules (18, 19). A number of studies show that TRPM2 channels, albeit with cell type-specific subcellular localization, serve widely as ROS-induced Ca^2+^ signaling mechanisms in the immune cells. The TRPM2 channels are located on the cell surface mediating Ca^2+^ influx in monocytes, macrophages, neutrophils, lymphocytes, and microglia, but preferentially present within the lysosomal membranes in DC for Ca^2+^ release. Efforts to investigate the role of the TRPM2 channels have been hampered by the lack of specific inhibitors (11). Recent studies, mainly using transgenic knockout (KO) mice and derived cells, have revealed that TRPM2 channels play an important role in numerous immune cell functions (Table 1) and TRPM2-mediated Ca^2+^ signaling mechanisms are crucial in many of these functions (Figure 1). Furthermore, studies combining with mouse models of various diseases have provided evidence to implicate the TRPM2 channels in the pathogenesis of numerous inflammatory diseases. This mini-review discusses the recent advances in understanding the roles of TRPM2 channels in immune cell functions and inflammatory diseases. Several recent reviews provide more information regarding the structural features, activation mechanisms, biophysical and pharmacological properties of TRPM2 channels, as well as their roles in excitable cells and other non-excitable cells (11, 20–23).

**Table 1:** **A summary of TRPM2 channel-dependent immune cell functions**.

Cell type	Associated cell functions	Reference
Monocyte	Production of CXCL2/CXCL8 induced by H_2_O_2_ and CXCL2 to in response to dextran sulfate sodium-induced colon inflammation	(24)
	LPS-induced production of IL-6, IL-8, IL-10, and TNF-α	(25)
	H_2_O_2_- and TNF-α-induced cell death	(14, 26)
Macrophage	Production of CXCL2 in response to carrageenan-induced inflammation or nerve injury and to LPS/IFN-γ	(27)
	Inhibition of LPS-induced production of ROS by NADPH oxidase	(28)
	Zymosan-induced production of CXCL2, G-CSF, and IL-1α	(29)
	Activation of NLRP3 inflammasome and IL-1β maturation induced by charged lipids, silica, and alum	(30)
	Up-regulated HO-1 expression induced by LPS, and cecal ligation and puncture	(31)
	H_2_O_2_-induced cell death	(32)
Microglia	Production of CXCL2 in response to nerve injury and LPS/IFN-γ	(27)
	LPS/IFN-γ-induced release of NO	(33)
Neutrophil	Sulfur mustard-induced priming and production of IL-6, IL-8, and TNF-α	(34)
	Adhesion of neutrophils to endothelial cells and myocardial infarction during reperfusion	(35)
	Migration of neutrophils and brain damage during reperfusion after ischemic stroke	(36)
Dendritic cell	Production of IL-12 in response to *Listeria monocytogenes*-induced infection	(37)
	Chemokine-induced cell maturation and migration, and chemotaxis to *E. coli*-induced infection	(38)

*CXCL, C-X-C ligand; LPS, lipopolysaccharide; ROS, reactive oxygen species; NADPH, nicotinamide adenine dinucleotide phosphate; IFN-γ, interferon γ; IL, interleukin; TNF-α, tumor necrosis factor-α; G-CSF, granulocyte colony stimulating factor; HO-1, heme oxygenase-1; NO; nitric oxide*.

## Production of Chemokine CXCL8/CXCL2

Human chemokine C-X-C motif ligand 8 (CXCL8) or mouse functional homolog CXCL2, produced by immune cells, plays an important role in recruiting neutrophils to the sites of infection and inflammation (39). For CXCL8/CXCL2 production by monocytes, ROS-induced Ca^2+^ influx is crucial in inducing the activation of extracellular-signal-regulated kinases (ERK) and ERK-dependent nuclear translocation of transcription factor nuclear factor-κB (NF-κB). In human U937 monocytic cells, H_2_O_2_-induced CXCL8 production was strongly dependent of extracellular Ca^2+^ and TRPM2 expression, and more specifically TRPM2-mediated Ca^2+^ influx was important in elevating the [Ca^2+^]_i_ as a signal for sequential activation of proline-rich tyrosine kinase (Pyk2), which is sensitive to Ca^2+^, Ras and ERK (24). Consistently, H_2_O_2_-induced Ca^2+^ influx and CXCL2 production were remarkably attenuated in monocytes from the TRPM2-KO mice. Migration of neutrophils from the wild-type (WT) mice was enhanced in culture medium preconditioned by H_2_O_2_-treated monocytes from the WT but not TRPM2-KO mice. CXCR2 is known to have a critical role in inducing ulcerative colitis (40). In response to dextran sulfate sodium (DSS)-induced colon inflammation, a model of ulcerative colitis, there was strong increase in the CXCL2 expression in monocytes from the WT but not TRPM2-KO mice (24). Neutrophil infiltration into the inflamed colons was increased in the WT mice, which was largely abolished in the TRPM2-KO mice. The TRPM2-KO mice manifested significantly reduced severity of colitis. Therefore, TRPM2-mediated Ca^2+^ influx is important in ROS-induced CXCL2/CXCL8 production by monocytes (Table 1 and Figure 1) and neutrophil infiltration that, if heightened to colon inflammation, lead to colitis.

Prominent CXCL2 production and neutrophil infiltration were also observed in the inflamed paw of the WT mice induced by carrageenan injection or in the site of nerve injury (27). Both CXCL2 production and neutrophil infiltration were significantly impaired in the TRPM2-KO mice. Nerve injuries can further elicit microglial activation in the spinal cord, which was clearly reduced in the TRPM2-KO mice. Lipopolysaccharide (LPS), found in Gram-negative bacterial walls, and interferon γ (IFN-γ), produced mainly by T lymphocytes and natural killer cells, are widely used in studying the immune responses to infection and inflammation, and both agents are known to stimulate the ROS production (41, 42). LPS/IFN-γ-induced CXCL2 production was reduced in macrophages and microglia from the TRPM2-KO mice (Table 1). The TRPM2-KO mice exhibited similar basal sensitivity to mechanical or thermal stimulation as the WT mice but reduced mechanical allodynia and thermal hyperalgesia after carrageenan-induced inflammation or nerve injury. These results provide consistent evidence to suggest that activation of the TRPM2 channels in macrophages and microglia in response to inflammation or nerve injury stimulates the CXCL2 production and neutrophil infiltration and thereby intensifies peripheral and spinal pro-nociceptive immune responses, leading to inflammatory and neuropathic pain.

Furthermore, three recent studies have examined the role of TRPM2 channels in the CXCL2 production by immune cells in responses to infection and there were significant discrepancies in these studies using different cell preparations and infection stimuli. The first study observed no difference between the WT and TRPM2-KO mice in the CXCL2 expression in splenocytes and neutrophil recruitment to the site of infection induced by injection of *Listeria monocytogense* (*Lm*), a model of listeriosis (37). In the second study, the CXCL2 production induced by zymosan, containing 1 → 3-β-glucans of fungal cell walls and known to induce ROS production, was significantly reduced in macrophages from the TRPM2-KO mice (29) (Table 1). Surprisingly, the third study reported an increase in the CXCL2 production in LPS-treated macrophages from the TRPM2-KO mice and also in the TRPM2-KO mice in response to LPS-induced lung inflammation (28), which is thought to result, as discussed further below, from increased NADPH oxidase activity and ROS production.

## Production of Proinflammatory Cytokines

Numerous proinflammatory cytokines are produced during the innate immune response to infection and inflammation. Numerous recent studies have investigated the role of the TRPM2 channels in the production of proinflammatory cytokines. LPS-induced production of IL-6, IL-8, IL-10, and tumor necrosis factor-α (TNF-α) in THP1 monocytic cells was significantly attenuated using short hairpin RNA (shRNA) to reduce the TRPM2 expression (25) (Table 1). Furthermore, LPS-induced Ca^2+^ influx and TNF-α generation were diminished upon removal of extracellular Ca^2+^ or after treatment with TRPM2 shRNA, supporting that TRPM2-mediated Ca^2+^ influx has a significant role (Figure 1). Zymosan-induced production of granulocyte colony-stimulating factor (G-CSF) and IL-1α was also strongly attenuated in macrophages from the TRPM2-KO mice (29) (Table 1). Sulfur mustard (SM), an alkylating agent used in chemical warfare, causes tissue damage and induces inflammatory responses. SM-induced production of IL-6, IL-8, and TNF-α by human neutrophils requires TRPM2-mediated Ca^2+^ influx to activate the p38 mitogen-activated protein kinase (p38 MAPK) signaling pathway (34) (Table 1 and Figure 1). By contrast, there was no difference in the IL-6 production by splenocytes and in the serum level of IL-6 between the WT and TRPM2-KO mice after *Lm* infection (37). The production of IL-6 and IL-10 in response to acute inflammation following ovalbumin/alum-induced severe allergy remained the same between the WT and TRPM2-KO mice (43). Zymosan-induced production of TNF-α in macrophages was not altered by TRPM2 deficiency (29). The production of IL-6 and TNF-α was however enhanced in LPS-treated macrophages from the TRPM2-KO mice and in response to LPS-induced infection in the TRPM2-KO mice (28). Evidently, further studies are required to clarify the noticeable discrepancies from these studies using different infection stimuli and cell preparations.

The production of IL-12 and IFN-γ after DSS-induced colon inflammation was significantly decreased in the TRPM2-KO mice (24). The production of these cytokines in *Lm*-treated splenocytes from the TRPM2-KO mice and the serum level of IL-12 and IFN-γ in the TRPM2-KO mice following *Lm* infection were also strongly reduced (37). Further analysis suggests that the TRPM2 channel function is required for the production of IL-12, the early inflammatory cytokine produced by DC (Table 1) and possibly other immune cells, which elicits IFN-γ-mediated innate immune responses. The deficient production of IL-12 and IFN-γ in the TRPM2-KO mice led to a significantly lower survival rate after *Lm* infection, supporting a vital role for the TRPM2 channel in the innate immune response to *Lm* infection (37).

As the residual macrophage in the CNS, microglia play a key role in the major immune responses to nerve damages by producing a number of proinflammatory mediators, including chemokine and nitric oxide (NO) (44–46). As discussed above, LPS/IFN-γ-induced CXCL2 production as part of the immune responses to peripheral nerve injury was strongly impaired in microglia from the TRPM2-KO mice (27). A recent study shows that LPS/IFN-γ-induced increase in the [Ca^2+^]_i_ and subsequent release of NO in microglia also depends on the TRPM2 channel function (33) (Table 1).

## Production of IL-1β

Immune cells such as macrophages and microglia produce IL-1β, a key proinflammatory cytokine in innate immunity (47). The production of the leaderless IL-1β (and also IL-18) optimally needs two signals termed the priming and activation signals. The priming signal stimulates a Toll-like receptor (TLR) such as TLR4 by LPS or other receptors to initiate signaling pathways leading to synthesis of pro-IL-1β. Activation or assembly of the NLRP3 inflammasome is required for activating caspase-1, which converts pro-IL-1β into IL-1β via proteolytic cleavage. A number of structurally diverse substances are known as the activation signal, including molecules released from damaged cells such as ATP, lipids, amyloid peptides, uric acid and mitochondrial DNA, environmental irritants like asbestos and silica, and alum used as a vaccine adjuvant (48–52). While ATP activates the NLRP3 inflammasome via the P2X7 receptor, the mechanisms for other activation signals remain less well understood. Accumulating evidence supports that many of them termed particulates can induce mitochondrial production of ROS but how the NLRP3 inflammasome is activated by ROS still remains a matter of extensive investigations (52). TRPM2 channels mediate Ca^2+^ influx as the major ROS-induced Ca^2+^ signaling mechanism in macrophages (32) (Figure 1). The NLRP3 inflammasome activation in macrophages by particulates such as charged lipids, silica, and alum was strongly dependent of extracellular Ca^2+^ and remarkably impaired in macrophages from the TRPM2-KO mice (30) (Figure 1 and Table 1). Thus, TRPM2-mediated Ca^2+^ influx is a critical step in coupling ROS generation to NLRP3 inflammasome activation and IL-1β maturation. It is unclear whether TRPM2-mediated Ca^2+^ signaling plays a similar role in the activation of NLRP3 inflammasome by other particulars that can induce ROS generation.

## Dendritic Cell Maturation and Chemotaxis

Dendritic cells play a critical role in presenting antigens to T lymphocytes and thus DC maturation and migration are crucial in linking the innate and adaptive immune responses (53). A recent study reveals that the TRPM2 channels preferentially function as a lysosomal Ca^2+^ release mechanism in DCs (38) (Figure 1). This study further showed that a high proportion of DCs from the TRPM2-KO mice exhibited significant reduction in chemokine-induced Ca^2+^ responses and loss of cell maturation (Table 1). Moreover, several chemokine receptors, including CXCR4, CXCR5, and CXCR7, were not up-regulated in the TRPM2-deficient DCs, and these cells failed to migrate to the site of infection induced by injection of *E. coli*. Therefore, TRPM2 channel-mediated lysosomal Ca^2+^ release provides the critical Ca^2+^ signal during DC maturation and chemotaxis.

## Post-Ischemic or Reperfusion Tissue Damages

Reperfusion is essential in preventing heart and brain damages induced by myocardial infarction and ischemic stroke, but it is well known that reperfusion results in excessive ROS production and causes additional tissue damage termed “reperfusion damage.” While numerous ischemia/reperfusion (I/R) damage mechanisms have been proposed [e.g., Ref. (23, 54)], oxidative stress-induced inflammatory response is strongly implicated in reperfusion damage. Consistently, myocardial infarction after I/R, but not ischemia alone, and post-ischemic cardiac contractile dysfunction were strongly reduced in the TRPM2-KO mice (35) (Table 1). Such protective results are dominantly, but not exclusively, due to reduced accumulation of neutrophils into myocardium during reperfusion. Moreover, the [Ca^2+^]_i_ in neutrophils, and neutrophil migration and adhesion to endothelial cells were enhanced by H_2_O_2_ in combination of leukotriene B_4_, an inflammatory mediator known to be involved in post-ischemic leukocyte infiltration. The increase in the [Ca^2+^]_i_ and adhesion were strongly attenuated, while migration remained unaltered in the TRPM2-deficient neutrophils (35). A recent study using TRPM2-KO mice has also demonstrated an important role for the TRPM2 channel in mediating brain damage after transient ischemia, but not ischemia without reperfusion (55). TRPM2 deficiency significantly reduced infarction and neurological deficits due to transient ischemia (36, 55, 56). Ischemic brain invasion by neutrophils and macrophages was noticeably reduced in the TRPM2-KO mice, suggesting a critical role for the TRPM2 channel in determining the migration of neutrophils and macrophages into ischemic brain tissues (36) (Table 1). These studies are consistent in supporting a detrimental role for post-ischemia ROS-induced activation of the TRPM2 channels in mediating inflammation that contribute to reperfusion damages after myocardial infarction and ischemic stroke.

## Regulation of Heme Oxygenase-1 Expression

Heme oxygenase-1 (HO-1) provides a protective mechanism by limiting oxidative stress-induced tissue damage during inflammation and sepsis (57, 58). A recent study has examined the role of TRPM2 channels in regulating HO-1 expression in sepsis using cecal ligation and puncture (CLP)-induced model (31) (Table 1). The HO-1 expression in mouse macrophages was enhanced by treatment with LPS *in vitro* and CLP *in vivo*. LPS-induced increases in the [Ca^2+^]_i_ and HO-1 expression were diminished by removing extracellular Ca^2+^ and in macrophages from the TRPM2-KO mice (Figure 1). CLP-induced increase in the HO-1 expression was also reduced in the TRPM2-KO mice. Furthermore, the TRPM2-KO mice exhibited significantly lower survival rate, accompanied with increased bacterial burden, tissue injury, and inflammation. Taken together, these results support the notion that TRPM2-mediated Ca^2+^ influx is important in up-regulating the HO-1 expression and enhancing bacterial clearance during sepsis.

## Modulation of NADPH Oxidase Activity

NADPH oxidases represent the primary mechanism by which phagocytes such as macrophages produce ROS during innate immune responses, and their enzymatic activity can be regulated by Ca^2+^, Ca^2+^-sensitive protein kinase C α (PKCα), and membrane potential (59). Similarly in response to *Lm* infection, the TRPM2-KO mice experienced a greater mortality than the WT mice after LPS-induced lung inflammation (28). However, the reduced survival as a result of TRPM2 deficiency in this case was due to increased ROS production in phagocytes (Table 1). Further detailed analysis reveals that Ca^2+^/Na^+^ influx and particularly K^+^ efflux mediated by the TRPM2 channels induces membrane depolarization and inhibits the membrane potential-sensitive NADPH oxidases (Figure 1). In conclusion, the TRPM2 channels in phagocytes provide a negative feedback mechanism, restricting NADPH oxidase-mediated ROS production, and thereby mitigating bacterial infection-induced lung damage.

## Cell Death

The most well-established role of the TRPM2 channels in diverse cell types is to mediate ROS-induced cell death (11). This was first demonstrated in a study using several cell types including U927 cells (14) (Table 1). Further examination showed that exposure to relatively low H_2_O_2_ concentrations (0.1 mM) induced substantial CXCL8 production but modest cell death in U937 cells (24). TNF-α can stimulate the ROS production (41). Treatment of U937 cells with TNF-α resulted in salient increase in the [Ca^2+^]_i_ and decrease in cell viability (26) (Table 1 and Figure 1). TNF-α-induced cell death was suppressed by using small interference RNA to reduce the TRPM2 expression or overexpressing TRPM2-S, an alternatively spliced and truncated isoform that cannot form a functional channel on its own but imposes dominant-negative inhibition of the activity of the TRPM2 channel formed by the full-length protein. Exposure to relatively high H_2_O_2_ concentrations (0.3–1 mM) strongly reduced the viability of RAW264.7 and macrophages from the WT mice and such cell death was reduced by using PJ-34, a PARP inhibitor, and also reduced by TRPM2 deficiency (32) (Table 1 and Figure 1). These findings are consistent with the idea that TRPM2 channels play an important role in the hierarchal model of oxidative stress (41), in which modest oxidative stress initiates intracellular signaling pathways for the production of inflammation proteins such as chemokines and cytokines whereas severe oxidative stress results in disruption in intracellular Ca^2+^ homeostasis and cell death.

## Concluding Remarks

Since the discovery of TRPM2 channels as ROS-activated Ca^2+^-permeable cationic channels in immune cells, studies have made important progress in better understanding their locations and functions in the immune cell functions. Studies in combination with mouse models have demonstrated the importance of the TRPM2 channels in immune cells at the system level; the TRPM2 channel activity is clearly crucial for both innate and adaptive immunity but excessive TRPM2 channel activity contributes significantly toward the pathogenesis of inflammatory diseases. The ongoing and future studies, with the aid of specific TRPM2 inhibitors that hopefully become available in the near future, will provide a more mechanistic insight into the TRPM2 channels in humans under physiological and pathological conditions that ultimately will enable us to explore the therapeutic potential of this amazing ion channel.

## Conflict of Interest Statement

The authors declare that the research was conducted in the absence of any commercial or financial relationships that could be construed as a potential conflict of interest.
